# Reward positivity and affective functioning in early childhood: evidence from temporospatial principal components analysis

**DOI:** 10.3389/fnhum.2026.1839910

**Published:** 2026-10-07

**Authors:** Yu Sun Chung, Armen Bagdasarov, Michael S. Gaffrey

**Affiliations:** 1 Department of Psychology and Neuroscience, Duke University, Durham, NC, United States; 2 Department of Psychology, Teen Experiences, Emotions and Neuroendocrinology Lab, Kean University, Union, NJ, United States; 3 Children’s Wisconsin, Milwaukee, WI, United States; 4 Division of Pediatric Psychology and Developmental Medicine, Department of Pediatrics, Medical College of Wisconsin, Milwaukee, WI, United States

**Keywords:** P200, positive affect, reward-related positivity, sex difference, temporospatial principal component analysis

## Abstract

Reward-related Positivity (RewP) indexes neural sensitivity to reward feedback and has been widely studied in adolescents and adults. However, little is known about how individual differences in RewP are associated with emotional functioning during early childhood, a critical period for affective development. Interpretation of feedback-related ERPs in children is further complicated by temporally overlapping components. This study examined whether a principal component analysis (PCA)-derived RewP is associated with affective functioning in children aged 4-7 years. Electroencephalography was recorded from 140 children during an age-appropriate reward task. Temporospatial PCA was performed on the full EEG sample (*n* = 140), and primary regression analyses were conducted in the subset with complete covariate data (*n* = 48). Temporospatial PCA was applied to isolate a RewP component differentiating gain from loss feedback. Positive and negative affect were assessed using the child version of the Positive and Negative Affect Schedule. Regression analyses examined whether PCA-derived RewP- and gain-related P200 amplitudes were associated with affective functioning while controlling for age, sex, ethnicity, income-to-need ratio, child depressive symptoms, and parental depressive symptoms. At the group level, boys showed greater PCA-derived gain-related P200 amplitudes than girls. At the individual level, higher child positive affect was associated with larger PCA-derived RewP amplitudes in the primary covariate-adjusted regression model. In sensitivity analyses conducted in the larger sample without the income-to-needs covariate, negative affect, rather than positive affect, was significantly associated with PCA-derived RewP amplitude. These findings provide novel evidence that RewP may serve as an early neural marker of affective functioning. By extending RewP research to an understudied developmental period and emphasizing normative emotional functioning, this study advances understanding of early reward processing and highlights the utility of PCA for isolating meaningful ERP components in children.

## 1 Introduction

Reward processing plays a central role in emotional development, motivation, and adaptive functioning across the lifespan. Many studies suggest that a neurobiologically based approach system regulates reward processing (Dillon et al., 2014) and generates positive emotions to facilitate and reinforce engagement with rewarding stimuli (Depue and Collins, 1999; Polak-Toste and Gunnar, 2006; Smillie et al., 2019; Wilt and Revelle, 2009). Neural sensitivity to reward outcomes is frequently examined using event-related potentials (ERPs), particularly the feedback-related negativity (FN) or reward positivity (RewP)—a component emerging 250–350 ms following feedback at frontocentral electrode sites reflecting differential responses to gains versus losses (Krigolson, 2018). In adolescents and adults, larger RewP amplitudes have been linked to greater positive affect, reward responsivity, and approach-related behavior, as well as lower risk for internalizing psychopathology (Amir et al., 2023; Brown et al., 2022; Jackson and Cavanagh, 2023; Proudfit, 2015). These findings position the RewP as a promising neural marker of reward-related affective functioning.

Despite growing interest in the developmental origins of reward processing, relatively little is known about how RewP relates to emotional functioning during early childhood. This period represents a critical window for affective development, characterized by rapid changes in emotional reactivity, regulation, and reward learning (Adynski et al., 2024; Hennefield et al., 2022; Montroy et al., 2016). Understanding neural correlates of positive affect during early childhood is particularly important given its associations with resilience, social competence, and long-term mental health (Forbes and Dahl, 2005; Hanson et al., 2021; Zhang et al., 2023). However, most ERP research has focused on adolescents or adults (Brown et al., 2022; Duttweiler et al., 2024; Irvin et al., 2022; Jackson and Cavanagh, 2023) and has primarily emphasized links between reward-related neural activity and psychopathology (Gibb, 2023; Kujawa, 2024; Proudfit, 2015) rather than normative variation in positive emotional functioning in young children.

Neurophysiological mechanisms supporting the evaluation of external feedback have long been studied using event-related potentials (ERPs), given their high temporal resolution and precise coupling between outcome onset and neural response. Early work focused on the feedback-related negativity (FN), a negative deflection occurring approximately 250–350 ms following feedback at frontocentral electrode sites (Miltner et al., 1997). However, more recent evidence suggests that the FN—often operationalized as the loss-minus-gain difference wave—may instead reflect a relative reduction in a RewP following non-reward outcomes (Heydari and Holroyd, 2016; Krigolson et al., 2014; Proudfit, 2015). This has led to the reconceptualization of the component as the RewP specifically evoked by positively valenced feedback. Despite this shift, debate remains regarding whether the observed signal reflects sensitivity to reward, loss, or both (Holroyd, 2004), as well as the extent to which it is influenced by temporally adjacent components such as the P200 and P300 (Krigolson, 2018). Nevertheless, the most recent dominant reinforcement learning account proposed by Cavanagh and Holroyd (2026) posits that the RewP indexes a dopaminergic positive reward prediction error signal (i.e., outcome that is better than expected), representing an abstract signal of goal attainment within mesocorticolimbic circuitry. Empirical evidence suggests that the RewP reflects a flexible neural signal of goal attainment: it is selectively enhanced by unexpected goal-congruent outcomes, can be elicited by abstract performance feedback, and is not limited to hedonic reward receipt, supporting its interpretation as a goal prediction error rather than a simple reward response (Cavanagh and Holroyd, 2026).

Studying feedback-related ERPs in early childhood presents unique methodological challenges because immature neural systems are characterized by longer response latencies, broader ERP morphology, and greater temporal overlap among adjacent components than those observed in older children and adults (Davies et al., 2010; de Haan, 2007; Hennefield et al., 2022; Lukie et al., 2014; Mueller et al., 2008; Rueda et al., 2004). In studies of older children, adolescents, and adults, the RewP has typically been quantified at frontocentral electrode sites (e.g., Fz, FCz, Cz) within windows spanning approximately 250–350 ms post-feedback, although the precise latency varies across developmental stages and analytic approaches (Bress et al., 2012; Kawamoto and Hiraki, 2019; Kessel et al., 2016; Kujawa et al., 2014; Moser et al., 2018). In contrast, studies focusing on young children have adopted broader and often later time windows (e.g., 250—up to 550 ms), sometimes incorporating more posterior recording sites (e.g., Pz), reflecting less temporally and spatially differentiated neural responses (Barch et al., 2020; Belden et al., 2016). For example, Hennefield et al. (2022) quantified the RewP in a large sample of 4–6-year-old children (*N* = 309) using a 250–500 ms window across multiple electrode sites (Fz, Cz, Pz, Oz). Consistent with these developmental characteristics, converging evidence indicates that the RewP in early childhood typically peaks later (approximately 300–500 ms at central recording sites) than in older children and adults (DeBoer et al., 2005; Brooker et al., 2020; de Haan, 2007; Webb et al., 2005). This delayed RewP frequently overlaps with neighboring components, particularly the P200 and P300, making it difficult to isolate reward-related neural activity using conventional ERP approaches. Consequently, applying adult-derived time windows to young children may obscure the functional interpretation of reward-related neural activity. These developmental characteristics are particularly relevant for the RewP, which overlaps both temporally and spatially with neighboring ERP components.

The examination of sex differences may be particularly relevant during early childhood, as emerging evidence suggests that males and females differ in attentional and emotional processing of motivationally salient information (Abbruzzese et al., 2019; Bourisly and Shuaib, 2018; van Hooff et al., 2011; Ycaza Herrera et al., 2019). Although sex differences in reward-related neural responses have been inconsistent across developmental studies (Ding et al., 2017; Greimel et al., 2018), earlier attentional components such as the P200 may show greater sex-related variability than later reward-evaluation processes (Aerts et al., 2015; Bourisly and Shuaib, 2018; Giannopoulos et al., 2022). Consequently, examining both P200 and RewP may help clarify whether sex differences emerge during early attentional allocation, later reward evaluation, or both.

The RewP is preceded by the P200, an early positive deflection peaking around 150–250 ms that has been linked to attentional allocation, arousal, and salience detection rather than reward valence (Carretié et al., 2001; Krigolson, 2018). Evidence suggests that the P200 reflects rapid attentional capture by motivationally relevant stimuli (Kotz and Paulmann, 2011) and may exhibit sex-related variability in attentional processing (Pfabigan et al., 2014; van Hooff et al., 2011; van Hooff et al., 2008), with some evidence of males having higher P200 following motivationally salient cues(van Hooff et al., 2011). The RewP is subsequently followed by the P300, a later positive component typically observed between approximately 250–500 ms that has been linked to attentional updating, evaluative processing, memory and context updating (Hajcak and Foti, 2020; Polich, 2007; Polich, 2012). Because these components occur in close temporal succession, conventional peak- or time-window-based ERP scoring approaches may conflate early attentional processes (P200), reward evaluation processes (RewP), and later outcome-updating processes (P300), potentially obscuring developmental and sex-related differences in reward responsiveness. Accordingly, temporospatial principal component analysis (PCA) provides a valuable approach for isolating overlapping neural processes and identifying developmentally meaningful variation in reward-related brain activity during early childhood.

A growing literature suggests that individual differences in positive affect are associated with enhanced neural responsiveness to reward, particularly as indexed by the RewP. Higher levels of positive affect and positive emotionality have been linked to larger RewP amplitudes, whereas reduced positive affect has been associated with blunted reward-related neural responses and increased vulnerability to depression (Brown et al., 2022; Duttweiler et al., 2024; Jackson and Cavanagh, 2023; Proudfit, 2015). However, most of this work has focused on adolescents and adults, and considerably less is known about how positive affect relates to reward-related neural activity during early childhood, a developmental period characterized by rapid maturation of both reward processing and positive emotional functioning. Moreover, little research has examined whether positive affect is associated with the feedback-elicited P200. Although affective and motivational states have been shown to modulate P200 amplitude, existing evidence primarily implicates the P200 in early attentional orienting toward salient information, and findings specifically linking positive affect to feedback-related P200 activity remain limited and mixed (Carretié et al., 2001; Kotz and Paulmann, 2011). Thus, by applying temporospatial PCA to separate the P200, RewP, and P300 in children aged 4–7 years, the present study extends prior research by examining whether positive affect is associated with distinct stages of feedback processing, including early attentional responses (P200), reward evaluation (RewP), and later outcome-updating processes (P300), in children aged 4–7 years.

Temporospatial principal component analysis (PCA) is well suited for addressing developmental challenges in ERP research by separating temporally overlapping neural activity into distinct temporal and spatial components (Kujawa et al., 2017; Lole et al., 2013). Unlike traditional scoring methods that rely on fixed latency windows derived largely from adult samples, PCA can more effectively distinguish early attentional processes (e.g., P200) from later reward-related activity (e.g., RewP) in young children, whose ERP components often show delayed latencies and greater overlap (Foti et al., 2011a). Applying temporospatial PCA in early childhood therefore provides a more precise characterization of the neural processes underlying salience detection and reward evaluation, improving measurement reliability and construct validity. This approach also enables examination of whether separable neural processes involved in reward processing show different associations with positive affect and whether theses associations vary by sex, given emerging evidence of sex-related differences in reward-related neural responses during development (Chaplin et al., 2023; Dhingra et al., 2021; Greimel et al., 2018).

Addressing this critical gap, the present study dissociates the RewP from temporally overlapping components using temporospatial PCA, isolating indices of early attentional allocation (P200) and later reward evaluation (RewP). We then examine sex-specific associations between these PCA-derived components of feedback processing-reflecting both early and later attentional processes- and positive affect in children aged 4–7 years. Neural responses to reward feedback were measured using a modified Doors Guessing Task (Bress et al., 2012; Gaffrey et al., 2018a). While prior work has largely focused on group-level sex differences in the FN/RewP (Kujawa et al., 2014; Moser et al., 2018), we specifically target PCA-derived indices of early attentional allocation to reward-related feedback, with a particular emphasis on the P200 component. This focus is motivated by evidence that positive and negative affect are closely tied to attentional bias and arousal (Liu et al., 2025), processes reliably indexed by the P200 (Han et al., 2014). Additionally emerging findings indicate sex differences in P200 amplitude and latency during emotional and cognitive processing (Bourisly and Shuaib, 2018). Sex differences in reward positivity (RewP) are generally modest and inconsistent in developmental samples (Bress et al., 2015; Crowley et al., 2013; Kujawa et al., 2014), whereas earlier attentional components such as the P200 may show somewhat greater sex-related variability (Aerts et al., 2015; Bourisly and Shuaib, 2018); however, in young children, developmental immaturity and component overlap likely account for more variance than sex (Bress et al., 2015; Proudfit, 2015). Thus, we hypothesize that positive affect would be associated with enhanced PCA-derived RewP responses to reward feedback, reflecting greater reward evaluation in children with higher positive emotionality. Given limited and mixed evidence for sex differences in the RewP during childhood, we did not predict robust sex differences in the RewP itself. However, because the P200 reflects early attentional allocation to motivationally salient feedback and prior studies suggest sex-related variability in P200 amplitude and latency, we explored whether boys and girls differed in PCA-derived P200 responses. We expected that sex differences, if present, would be more evident in the P200 than in the RewP, reflecting variability in early attentional engagement with feedback rather than later reward evaluation. Accordingly, we hypothesized that (1) higher positive affect would be associated with larger PCA-derived RewP amplitudes, (2) positive affect would show weaker or less consistent associations with the P200 and P300, and (3) sex differences, if present, would be more evident in the P200 than in the RewP.

## 2 Methods and materials

### 2.1 Participants

The current study included a total of one hundred forty children between the ages of four and seven. Children were recruited as part of a larger longitudinal study investigating relationships between neural reward processing and early emotional development in young children with and without risk for depression (Camacho and Gaffrey, 2026). Additional details about participant recruitment and exclusion criteria are provided in the Supplementary material. For the current analyses, we focused on EEG reward processing data collected at baseline along with relevant individual difference measures. Prior to the primary analyses, multivariate outliers were identified using Mahalanobis distance (Tabachnick and Fidell, 2019). Consistent with conventional recommendations, cases exceeding the critical chi-square value corresponding to *p* < 0.001 were considered multivariate outliers. Because Mahalanobis distance is calculated based on the predictors included in each model, outlier screening was initially conducted separately for each hierarchical regression model. To ensure a consistent analytic sample across models, all cases identified as multivariate outliers in any regression model were excluded from all primary regression analyses (*n* = 6). Income-to-needs ratio data were available for a subset of participants (*n* = 48). Because hierarchical regression analyses were conducted using listwise deletion, models including income-to-needs ratio were restricted to participants with complete data on all predictors and outcome variables. In contrast, the temporospatial PCA was conducted on the full EEG sample (*n* = 134 after EEG quality-control exclusions) to maximize the stability of the component decomposition. PCA factor scores were then extracted for each participant and used in the hierarchical regression analyses. Consequently, the PCA decomposition was derived from the larger EEG sample, whereas hypothesis testing was conducted in the subset of participants with complete behavioral and demographic data (*n* = 48).

### 2.2 Behavioral and diagnostic measures

#### 2.2.1 Income-to-needs ratio

It was included as a covariate (White et al., 2022) in regression analyses by operationalizing it as the total family income divided by the federal poverty level based on a family size (McLoyd, 1998).

#### 2.2.2 Preschool Feelings Checklist-Scale Version (PFC-S)

The PFC-S (Luby et al., 1999) is a 23 item measure that uses a Likert rating scale (0 = never, 4 = most of time) and was used as the primary dimensional measure of depressive symptom severity in children and was included as a covariate in regression analyses (Gaffrey et al., 2018a). The measure has been validated for use in children aged 3-7-year-olds (Luby et al., 2012; Luby et al., 2004) and consists of 23 items adapted from the original Preschool Feelings Checklist (Luby et al., 2004). Caregivers rated the presence and severity of depressive symptoms, with higher scores indicating greater depression severity. Internal consistency in the current sample was good (Cronbach’s *α* = 0.80, *n* = 130 due to four participants with at least one item missing).

#### 2.2.3 Beck Depression Inventory-II (BDI-II)

The BDI-II (Beck et al., 1996) is a well-used and well-validated 21-item self-report inventory of depressive symptomatology in adults. Because parental depression may inflate parent-reported child’s negative affect and psychopathology symptoms (Bouvette-Turcot et al., 2020; Merwin et al., 2017), caregiver depressive symptoms were included as a covariate in primary analyses. Respondents rated each item on a 4-point scale reflecting symptom severity over the past 2 weeks, with higher scores indicating greater depressive symptomatology. Internal consistency in the current sample was good (Cronbach’s *α* = 0.87, *n* = 134).

#### 2.2.4 Positive and Negative Affect Schedule for Children (PANAS-C)

Child affect was assessed using a caregiver-report version of the PANAS that has been previously used with young children (Allan et al., 2013; Camacho et al., 2025; Hughes and Kendall, 2009; Laurent et al., 1999). Caregivers rated the extent to which their child experienced positive affect (e.g., excited, attentive, active) and negative affect (e.g., afraid, ashamed, hostile) during the previous week using a 5-point Likert scale ranging from 1 (Very slightly or not at all) to 5 (Extremely). Positive and negative affect subscale scores were computed by summing the relevant items. Internal consistency in the current sample was excellent for Positive Affect (α = 0.91) and good for Negative Affect (α = 0.82).

### 2.3 EEG task and materials

A developmentally sensitive version of the Doors Guessing Task was used to assess neural responses to reward and loss feedback using EEG method (Gaffrey et al., 2018a). The task consisted of 90 trials, including 30 gain, 30 loss, and 30 neutral trials presented in a predetermined pseudorandom sequence. Consistent with the conventional operationalization of the RewP in the Doors task literature, the present analyses focused on the gain-versus-loss contrast to isolate reward-related neural responses. Although neutral feedback trials were included to complete the task structure, they were not used to quantify the RewP because the neutral feedback (“--”) differed perceptually from the gain and loss feedback, which consisted of candy images. Consequently, gain–neutral and loss–neutral comparisons would be difficult to interpret, as observed differences could reflect perceptual as well as reward-related processes. On each trial children viewed two doors, with one randomly selected to have a button image on it (see Figure 1). To reduce cognitive and motor demands and maximize task feasibility for young children, a single-button response device was used rather than requiring separate responses for each door. Children were instructed to push the button if they wanted to select the door with the button image on it and to not push the button if they wanted to select the other door (i.e., the one without the button image on it). After their repones (i.e., button press or 2,000 ms pass), children received feedback indicating either a reward (four candies), a loss (two candies crossed out with red lines), or a neutral outcome (two dashes, “--”). Consistent with prior developmental adaptations of reward processing tasks (Gaffrey et al., 2018a), feedback outcomes were independent of participants’ responses and were presented according to the predetermined pseudorandom sequence, ensuring equal numbers of gain, loss, and neutral trials and preventing extended runs of the same outcome (i.e., no more than three in a row of the same outcome) for ERP analyses. Candy incentives were used instead of money rewards because they are more developmentally meaningful for young children (Gaffrey et al., 2018a; Luking et al., 2014). The task was administered using E-Prime (Psychology Software Tools, Inc., Sharpsburg, PA). Analyses focused on neural responses to gain and loss feedback, particularly the Reward Positivity (RewP), rather than behavioral choice performance.

**Figure 1:**
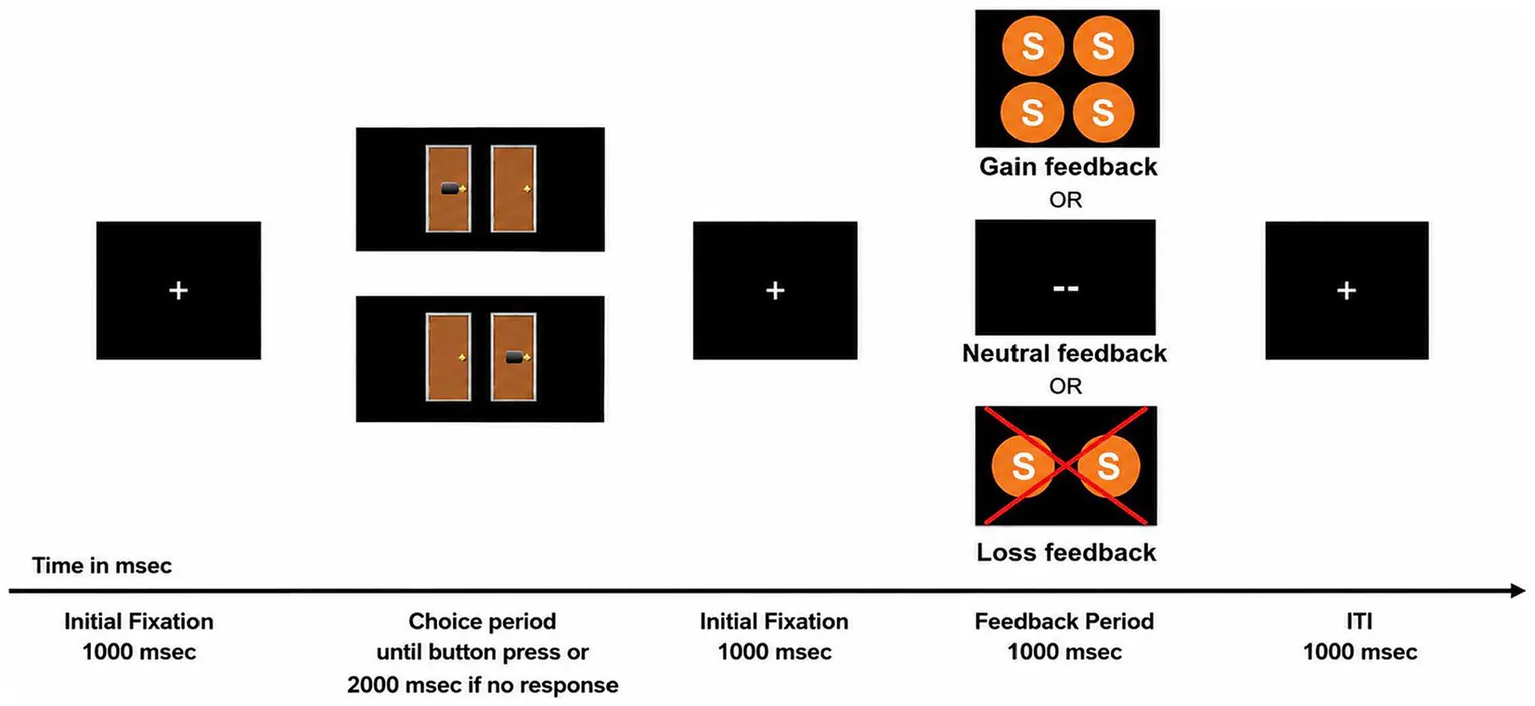
Schematic of the Modified Doors Task Trial. We focused on gain and loss feedback only due to different physiological features of neutral feedback in present study. Each trial began with a 1,000-ms fixation cross followed by a choice period during which children selected one of two doors using a single-button response. During the choice period, a black marker was randomly presented on one of the doors, children were told that pushing the button selected that door and not pushing it selected the other. After a button push or 2,000-ms elapsed a second 1,000-ms fixation period followed, after which gain, neutral, or loss feedback was presented for 1,000 ms. Feedback consisted of three outcome types: Gain feedback (four candies), neutral feedback (two dashes, “--”), and loss feedback (two candies crossed out with red lines). Outcomes were pseudorandomized and independent of participants’ choices. Gain, neutral, and loss outcomes were presented in equal proportions and arranged in a pseudorandom sequence to prevent extended runs of more than three of the same outcome type in a row.

### 2.4 EEG data acquisition and preprocessing

Continuous EEG was recorded using a 128-channel HydroCel Geodesic Sensor Net (Electrical Geodesics, Eugene, OR) and Net Amps 400 series amplifiers at a sampling rate of a 1,000 Hertz (Hz). Data was referenced online to the vertex (Cz), and impedances were maintained below 50 kilohms throughout the task paradigm.

Offline EEG data preprocessing was performed using EEGLAB version 2021 (Delorme and Makeig, 2004) in MATLAB R2019b (The MathWorks Inc., Natick, MA) using custom scripts available on https://github.com/gaffreylab. Like our recent work Bagdasarov et al. (2024) and Chung et al. (2025), we followed EEG preprocessing pipeline recommended for pediatric populations by (Debnath et al., 2020). Detailed steps are provided in the Supplementary material and in custom scripts available on https://github.com/gaffreylab. Briefly, channels located on the outer ring of the sensor net were removed. Data were then down sampled to 250 Hz, low-pass filtered at 40 Hz, and high-pass filtered at 1 Hz. Remaining electrical line noise at 60 Hz was attenuated using the CleanLine plugin (Mullen, 2012). Bad channels were removed by running the Clean Rawdata plugin; a channel was considered bad if (1) it was flat for more than 5 s, (2) contained more than four standard deviations of line noise relative to its signal, or (3) correlated at <0.8 to nearby channels. Artifact Subspace Reconstruction (ASR; Mullen et al., 2015) removed large artifacts with Clean Rawdata. Extended infomax independent component analysis (ICA; Lee et al., 1999) with PCA dimension reduction (50 components) was performed, and the resulting ICA matrix was then copied over to the original full-length data (i.e., before ASR removed artifacted portions). The ICLabel plugin (Pion-Tonachini et al., 2019) automatically removed independent components with a probability greater than 0.7 of being associated with eye movements and blinks. The data were segmented for each gain and loss trial, beginning 500 ms before feedback onset and continuous for 800 ms following feedback onset. Baseline correction was applied to the 200 ms window before feedback onset. Artifact rejection using the Trial-By-Trial plugin (Ben-Shachar, 2018) removed epochs with at least 10 channels meeting the following criteria: (1) peak-to-peak amplitudes exceeding 100 μV within 200 ms windows sliding by 20 ms, (2) voltages less than −150 or greater than +150 μV, or (3) joint probabilities above three standard deviations for local and global thresholds. If less than 10 channels in an epoch met criteria for rejection, the epoch was not removed, but the identified channels were interpolated for that epoch only. Lastly, the averaged ERPs for each bin (i.e., gain, loss) were computed. Following preprocessing, participants retained an average of 24.48 gain trials (SD = 5.25; 82% retention; 18.4% removed; range = 6–30 trials) and 23.97 loss trials (SD = 5.56; 80% retention; 20.1% removed; range = 5–30 trials).

### 2.5 Temporospatial principal components analysis (PCA)

Temporospatial PCA was conducted on the full EEG sample (*n* = 134 after quality-control exclusions) to obtain a stable decomposition of feedback-related ERP components. PCA factor scores were subsequently extracted for each participant and used in the hierarchical regression analyses. Because the primary regression models included income-to-needs ratio as a covariate, only participants with complete covariate and ERP data (*n* = 48) were included in those analyses.

Previous research suggests that the RewP/FN overlaps with other reward-related ERP components, including the P200 and P300 (Foti et al., 2011a; Proudfit, 2015; Schaefer et al., 2016; Schuermann et al., 2012). PCA has been shown to effectively decompose ERP waveforms into their underlying temporal and spatial components, facilitating the isolation of overlapping neural processes (Kayser and Tenke, 2003). Following prior recommendations for ERP PCA (Dien, 2010a; Dien et al., 2007) and previous applications to reward-processing ERPs (Foti et al., 2011a), temporospatial PCA was conducted on baseline-corrected ERPs separately averaged for gain and loss trials.

The analysis was conducted using the ERP PCA Toolkit (Version 2.93; Dien, 2010b). First, a temporal PCA was performed to capture variance across time points. Promax rotation was applied, and 18 temporal factors were retained based on visual inspection of the Scree plot using the elbow criterion (Cattell, 1966), whereby factors preceding the point at which the eigenvalue curve substantially leveled off were retained. For each temporal factor, factor scores were generated for each participant, electrode, and trial type, representing the amount of variance in the original ERP data accounted for by that factor.

Next, the spatial distribution of each temporal factor was analyzed using spatial PCA. Separate spatial PCAs were conducted for each of the 18 temporal factors using Infomax rotation. Based on visual inspection of the averaged Scree plots across temporal factors, five spatial factors were retained for each temporal factor using the elbow criterion, resulting in 90 temporospatial factor combinations (18 temporal × 5 spatial factors). The covariance matrix and Kaiser normalization were used for both temporal and spatial PCA procedures. Finally, factor waveforms were reconstructed in microvolts by multiplying the factor pattern matrix by the corresponding standard deviations. Consistent with prior ERP PCA studies (Foti et al., 2011a), the temporospatial factor corresponding to the RewP/FN was identified based on its temporal characteristics, scalp topography, and sensitivity to gain versus loss feedback.

### 2.6 Statistical analyses

Statistical analyses were conducted using IBM SPSS Statistics (IBM Corp, Armonk, NY; Version 31). The FN and RewP were defined as the mean amplitude between 344 and 444 ms following feedback indicating loss or gain, respectively, from each PCA factor representing the ERP of interest. The identified mean amplitudes from loss or gain for each individual were subsequently exported to SPSS for use in linear regressions.

Because gain- and loss-related ERP amplitudes are substantially correlated and contain shared variance, residualized scores were computed to isolate neural activity uniquely associated with responses to gain or loss outcomes. This approach provides a more specific index of reward-related processing than raw difference scores alone (Luking et al., 2017; Meyer et al., 2017). Specifically, loss-related activity was residualized by regressing FN on RewP and saving the unstandardized residuals (_resid_ FN), whereas gain-related activity was residualized by regressing RewP on FN and saving the unstandardized residuals (_resid_ RewP). Similarly, residualized scores for the P200 component were calculated by regressing P200 amplitudes on loss- or gain-related activity and saving the unstandardized residuals (_resid_ P200). These residualized measures were used in the primary analyses. Similarly, P200 and P300 were also examined as residualized gain scores. The mean amplitude of the P200 was measured between 192 and 292 ms following feedback, whereas the same 344–444 ms time window used for the RewP/FN was used to quantify the P300. To characterize the reward sensitivity of each PCA-derived factor, gain and loss amplitudes were compared using two-tailed paired-samples *t*-tests. Effect sizes were calculated as Cohen’s *d* for repeated-measures comparisons. Residualization was particularly useful in the present study because temporospatial PCA yields component scores that may retain variance shared across gain and loss conditions, and the residualized approach facilitates interpretation of outcome-specific neural responses.

Sex differences in age, parental depression, and child depression and negative and positive affect scores were examined using independent sample *t*-tests (see Table 1). Hierarchical regression analyses were conducted to examine whether child positive affect and negative affect predicted PCA-derived P200 and RewP amplitudes related to gain feedback while controlling for child age, ethnicity, income-to-needs ratio, parental depressive symptoms, child depressive symptoms, and sex. Overall model fit and block-level F-change statistics were evaluated using an alpha level of 0.05. Because the primary hypotheses concerned the associations between child affect and two ERP outcomes (P200 and RewP), statistical significance of the regression coefficients for the affect predictors was additionally evaluated using a Bonferroni-corrected threshold of *p* < 0.025 (0.05/2). Under this corrected threshold, the association between child positive affect and RewP remained significant. Due to missing income-to-needs ratio data, hierarchical regression analyses were conducted on the subset of participants with complete data (*n* = 48). Sex was included as a categorical predictor and was effect coded (female = −0.5, male = 0.5) in all regression models. Because positive affect and negative affect were specified *a priori* as theoretically motivated predictors, statistical significance was evaluated using an alpha level of 0.05. To address concerns regarding multiple testing across the two primary regression models, results were also evaluated using a Bonferroni-corrected significance threshold of *p* < 0.025 (0.05/2). Standardized regression coefficients (β) are reported for all hierarchical regression analyses. Sensitivity analyses were conducted to evaluate the robustness of findings to trial-count thresholds and model specification.

**Table 1:** Participants characteristics.

Variables	Participants (*n* = 134)		Sex difference
	Female (*n* = 70)	Male (*n* = 64)	*t*-test (133), *p*-value
Child age (months)	73.41 (± 12.16)	70.61 (±10.55)	−1.41, *p* = 0.15
Income-to-needs ratio (*n* = 48)	3.19 (± 1.00)	2.89 (± 1.18)	−1.56, *p* = 0.11
Ethnicity			Chi-square (1) = 0.13, *p* = 0.78
Hispanic or Latino	*N* = 6	*N* = 7	
Not Hispanic or Latino	*N* = 64	*N* = 57	
Race			Chi-square (4) = 2.49, *p* = 0.64
White	*N* = 55	*N* = 46	
African American	*N* = 4	*N* = 4	
Asian	*N* = 0	*N* = 2	
Mixed	*N* = 9	*N* = 9	
Other	*N* = 2	*N* = 3	
Individual differences			
Parental Beck Depression Inventory	3.95 (± 4.86)	5.47 (± 5.52)	1.65, *p* = 0.10
Preschool Feeling Checklist (PFC)	11.81 (± 6.24)	12.58 (± 7.86)	0.63, *p* = 0.52
Child positive affect^a^	33.43 (±7.17)	36.2 (±7.17)	2.21, *p* = 0.03*, Cohen’s *d* = 0.38
Child negative affect^a^	14.08 (±3.68)	15.41 (±4.50)	1.85, *p* = 0.07

^a^Child positive and negative affect were calculated as sum scores of items from the Positive Affect and Negative Affect subscales of the Positive and Negative Affect Schedule-Child version (PANAS-C)^1^. Values are presented as mean (SD) unless otherwise noted. Sample sizes vary across variables because of missing data. Income-to-needs ratio data were available for a subset of participants (*n* = 48), whereas ERP, demographic, and questionnaire measures were available for most participants. Child positive and negative affect were calculated as sum scores of items from the Positive Affect and Negative Affect subscales of the Positive and Negative Affect Schedule–Child Version (PANAS-C). *p < 0.05.

## 3 Results

As presented in Table 1, males and females did not show any sex differences in age and other individual difference measures of depression, except child positive affect: Males showed greater positive affect scores compared to females (*t*(130) = 2.21, *p* = 0.03).

### 3.1 The grand average waveforms before PCA

Waveforms depicting the grand average ERPs (i.e., before PCA) are presented in Figure 2. Consistent with prior work with adults (Foti et al., 2011b; Proudfit, 2015), our data showed the presence of FN (i.e., loss-gain) or RewP (i.e., gain-loss) at frontocentral electrodes including E19 in young children. In addition, and again consistent with prior work in older age groups, we confirmed that the deflection in the waveform for gain minus loss was superimposed on the P300 at ~400 ms.

**Figure 2:**
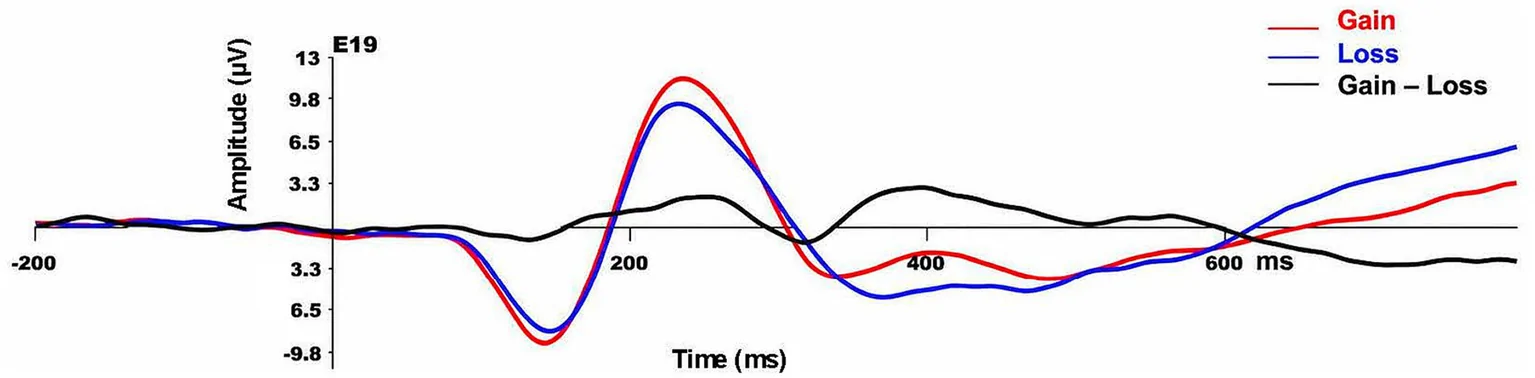
Grand-average reward-related ERP waveforms prior to PCA decomposition. Grand-average ERP waveforms at electrode E19 (corresponding to left Fz) for gain outcomes (red), loss outcomes (blue), and the Gain − Loss difference waveform (black) across all participants. Waveforms represent the original averaged EEG data prior to temporospatial principal component analysis (PCA). Positive values are plotted upward. The difference waveform illustrates reward-related neural activity by highlighting differential responses to gain versus loss feedback. See Supplementary Figure S1 for the full 128-channel HydroCel Geodesic Sensor Net.

When we examined sex differences in gain-related RewP or loss-related FN amplitudes *before* the PCA method, no sex differences were observed [*t*(131) = 1.30, *p* = 0.19; *t*(131) = −1.30, *p* = 0.20, respectively].

### 3.2 The temporospatial PCA results

Of the 90 total factor combinations yielded by the PCA, 18 accounted for at least 1% of the total variance in the data. Consistent with prior work (Dien and Frishkoff, 2005), visual inspection of the waveforms associated with these 18 factors was used to select those that most readily corresponded to ERP components relevant to the current task paradigm. Three temporospatial PCA factor combinations were selected for further statistical analyses, corresponding to the P200, RewP, and P300 components (see Table 2; Figures 3, 4 for the PCA-derived waveforms and scalp topographies).

**Table 2:** PCA factor combinations selected for statistical analysis.

Corresponding ERP component	Temporospatial factor combination	Variance explained (%)	Temporal loading peak (ms)	Spatial distribution	Peak channel	Gain (mean SD)	Loss (mean SD)	Gain-Loss *t*(132)	Cohen’s *d*
P200	TF4/SF1	3.7	240	Fronto-central positivity	EEG 6	6.62 (5.07)	4.93 (5.07)	4.93***	0.43
Rew positivity	TF1/SF1	15	392	Fronto-central negativity	EEG 19	−2.20 (8.36)	−3.48 (7.82)	2.08*	0.18
P300	TF1/SF2	5.4	392	Centroparietal positivity	EEG 71	6.65 (6.01)	7.40 (6.77)	−1.44	−0.12

PCA = principal component analysis; ERP = event-related potential. Temporal loading peak refers to the latency at which the temporal factor reached its maximum loading. Gain-loss comparisons were evaluated using paired-samples *t*-tests. Cohen’s *d* represents the standardized effect size for paired-samples comparisons of gain and loss conditions. For all factors, positive t values indicate that gain responses were more positive (or less negative) than loss responses, regardless of the absolute polarity of the PCA-derived component. *p = 0.01, ***p < 0.001.

**Figure 3:**
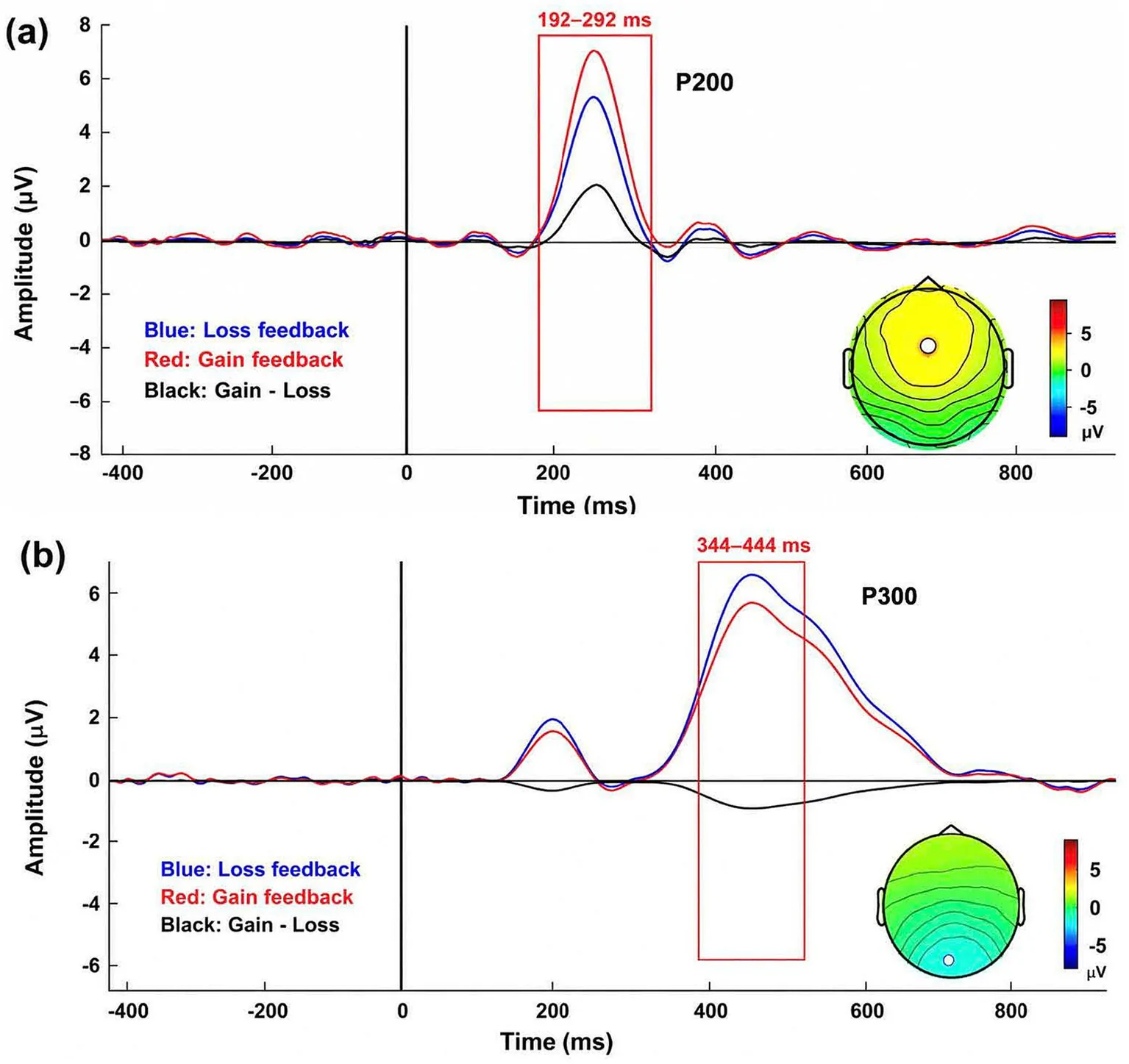
PCA-derived P200 and P300 ERP components. **(a)** ERP waveforms recorded at EEG electrode 6, shown separately for loss feedback (blue), and their difference (gain minus loss, black). The red box indicates the time window used for statistical analysis of the P200 component (192–292 ms). The topography in the lower right corner depicts the scalp map of the gain-loss difference at the temporal peak (240 ms). The white dot indicates the location of EEG 6. **(b)** ERP waveforms recorded at EEG electrode 71, shown separately for loss feedback (blue), gain feedback (red), and their difference (gain minus loss, black). The red box indicates the time window used for statistical analysis of the P300 component (344–444 ms). The topography in the lower right corner depicts the scalp map of the gain-loss difference (gain minus loss) at the temporal loading peak (392 ms). The white dot indicates the location of EEG 71.

**Figure 4:**
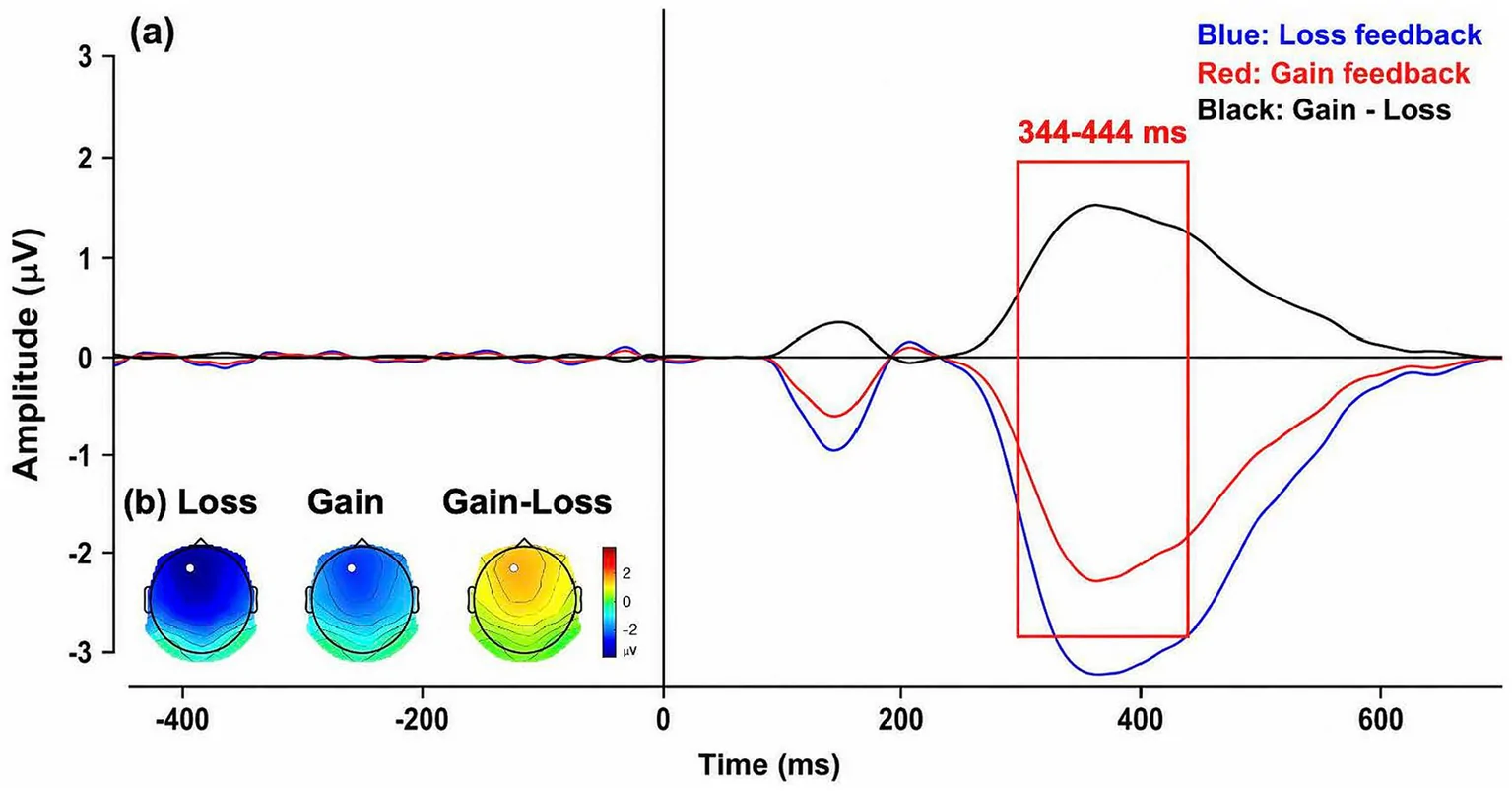
PCA-derived RewP component. **(a)** The waveform at EEG19 represents the portion of the ERP associated with TF1/SF1, the temporospatial PCA factor corresponding to the Reward Positivity (RewP) component. Waveforms are shown separately for gain outcomes (red), loss outcomes (blue), and the Gain − Loss difference waveform (black). Positive amplitudes are plotted upward. **(b)** Scalp topographies for loss feedback, gain feedback, and the Gain − Loss difference waveform during the 344–444 ms time window, when the temporal loading of TF1/SF1 was maximal. The loss condition exhibited greater fronto-central negativity, whereas the gain condition showed relatively reduced negativity. The Gain − Loss difference topography revealed a fronto-central positivity characteristic of the RewP. The white dot indicates the location of EEG19, the peak site of the PCA-derived RewP component. See Supplementary Figure S1 for the full 128-channel HydroCel Geodesic Sensor Net.

The waveforms and scalp topography associated with Temporal Factor 4, Spatial Factor 1 (TF4/SF1), the PCA factor corresponding to the P200 is presented in Figure 3a. As evident in the grand-average PCA-derived waveforms, this PCA factor represents a relative positivity for gain compared to loss trials that was maximal at frontocentral sites, with a peak at EEG 19. The gain–loss difference was statistically significant and small in magnitude (*d* = 0.18; Table 2). Interestingly, the PCA waveform revealed negative responses to both gain and loss trials, with the less negative response for gain resulting in a relative positivity compared with loss. The waveforms and scalp topography associated with Temporal Factor 1, Spatial Factor 2 (TF1/SF2), corresponding to the P300, are presented in Figure 3b. This factor was maximal at centroparietal sites, with a peak at EEG 71. As shown in the grand-average PCA-derived waveforms, gain and loss outcomes elicited similar amplitudes. The gain–loss difference was small in magnitude and did not reach statistical significance, *t*(132) = −1.44, *p* = 0.15, *d* = −0.12 (Table 2). Therefore, the P300 was not included in subsequent regression analyses. Lastly, the waveforms and scalp topography associated with Temporal Factor 1, Spatial Factor 1 (TF1/SF1), the PCA factor corresponding to the RewP (i.e., gain-loss) is presented in Figure 4. As evident in the grand average ERP waveforms of the PCA, this PCA factor represents a relative positivity for gain compared to loss trials that is maximal at frontocentral sites with a peak site of EEG 19, with the difference across feedback types being statistically significant (Table 2). Interestingly, the PCA waveform reveals negative responses to both gain and loss trials, with the less negative response for gain resulting in a positivity when compared to loss.

### 3.3 The temporospatial PCA revealed sex differences in P200, but not RewP ERP components

We conducted independent *t*-tests to examine sex differences in PCA-driven ERP components showing significant differences in neural responses during gain versus loss (i.e., using each temporal and spatial factor combinations generated from the PCA). Different from expectation, there were no sex differences in gain-related _resid_ RewP amplitudes from T1/SF1 factor combination, [(*t*)128) = 1.47, *p* = 0.14]. However, as expected, regarding PCA-driven P200, males showed greater gain-related _resid_ P200 from TF4SF1 [(*t*(131) = 3.32, *p* = 0.001, Cohen’s *d* = 0.57] compared to females.

### 3.4 Hierarchical regression for child positive affect predicting PCA-derived _resid_ P200 and _resid_ RewP

Hierarchical linear regression models were conducted to examine associations of child positive or negative affect with PCA-derived _resid_ P200 and _resid_ RewP after adjustment for child age, ethnicity, income-to-needs ratio, sex, parental depressive symptoms, and child depression symptoms. Across both models, Step 1 covariates did not significantly account for variance in the outcomes (*p*s > 0.15).

For PCA-derived _resid_ P200 relating to gain, inclusion of sex in Step 2 significantly improved model fit, *ΔR^2^* = 0.14, *F*-change (1,41) = 7.43, *p* = 0.009. Sex was a significant predictor of P200 amplitude (*β* = 0.40, *t* (41) = 2.72, *p* = 0.009), with boys exhibiting larger gain-related P200 amplitudes than girls. This effect remained significant after Bonferroni correction across the two primary regression models (adjusted α = 0.025). Addition of positive affect and negative affect in Step 3 did not significantly improve model fit, *ΔR^2^* = 0.05, *F*-change (2,39) = 1.28, *p* = 0.29. Neither positive affect (*β* = 0.22, *t* (39) = 1.36, *p* = 0.18) nor negative affect (*β* = 0.12, *t*(39) = 0.70, *p* = 0.48) was significantly associated with P200 amplitude (see Table 3).

**Table 3:** Summary of linear regression analysis for child positive affect predicting PCA-derived _resid_ P200 relating to gain.

Step	Variable	*B*	*SE B*	*β*	*t*	Significance	*R^2^* change	Sig. *F* change
Step 1							0.11	0.37
	Child age	−0.05	0.04	−0.19	−1.23	0.22		
	Ethnicity	0.20	0.34	0.08	0.594	0.55		
	Income-to-needs ratio	−0.35	0.50	−0.11	−0.70	0.48		
	Parental BDI-II	0.13	0.09	0.24	1.43	0.15		
	Child PFC depression	−0.07	0.07	−0.17	−1.10	0.27		
Step 2							0.13	0.009
	Sex	2.41	0.88	0.40	2.72	0.009**		
Step 3							0.04	0.28
	Child positive affect	0.09	0.06	0.22	1.36	0.18		
	Child negative affect	0.09	0.13	0.12	0.70	0.48		

Child positive and negative affect were calculated as sum scores of items from the Positive Affect and Negative Affect subscales of the Positive and Negative Affect Schedule-Child version (PANAS-C). B = unstandardized regression coefficient; β = standardized regression coefficient; SE = standard error; BDI = Beck Depression Inventory-II; PFC = Preschool Feelings Checklist-Scale Version. **p < 0.01.

As presented in Table 4, for PCA-derived _resid_ RewP, inclusion of sex in Step 2 did not significantly improve model fit (*ΔR^2^* = 0.00, *F*-change (1,41) = 0.00, *p* = 0.99). Inclusion of child positive affect and negative affect in Step 3 significantly increased explained variance (*ΔR^2^* = 0.12, *F*-change (2,39) = 3.31, *p* = 0.04). Within the final model, positive affect was a significant predictor of the _resid_ RewP relating to gain (*β* = 0.40, *t*(39) = 2.35, *p* = 0.02), and this effect remained significant following a Bonferroni-corrected significance threshold of *p* < 0.025 (0.05/2); see Figure 5. In contrast, negative affect was not a significant predictor of the RewP (*β* = 0.16, *t* (39) = 0.80, *p* = 0.43).

**Table 4:** The linear regression analysis for variables predicting PCA-derived RewP relating to gain and positive affect in young children.

Step	Variable	*B*	*SE B*	*β*	*t*	Significance	*R^2^* change	Sig. *F* change
Step 1							0.05	0.80
	Child age	0.06	0.07	0.13	0.86	0.39		
	Ethnicity	0.02	0.54	0.006	0.04	0.96		
	Income-to-needs ratio	−0.24	0.79	−0.05	−0.31	0.75		
	Parental BDI	0.003	0.15	0.003	0.01	0.98		
	Child PFC depression	−0.09	0.11	−0.13	−0.82	0.41		
Step 2							0.00	0.98
	Sex	0.02	1.51	0.003	0.01	0.98		
Step 3							0.12	0.04*
	Child positive affect^a^	0.26	0.11	0.40	2.35	0.02^b^		
	Child negative affect^a^	0.17	0.22	0.15	0.80	0.42		

*p < 0.05. ^a^The Positive Affect is sum score from the Positive and Negative Affect Schedule-Child Version. ^b^Child positive affect and child negative affect were the primary predictors of interest. Overall model and F-change statistics were evaluated at α = 0.05. To account for multiple testing across the two primary ERP regression models (PCA-derived P200 and PCA-derived RewP), the regression coefficients for the affect predictors were additionally evaluated using a Bonferroni-corrected significance threshold of *p* < 0.025 (0.05/2). B = unstandardized regression coefficient; β = standardized regression coefficient; SE = standard error.

**Figure 5:**
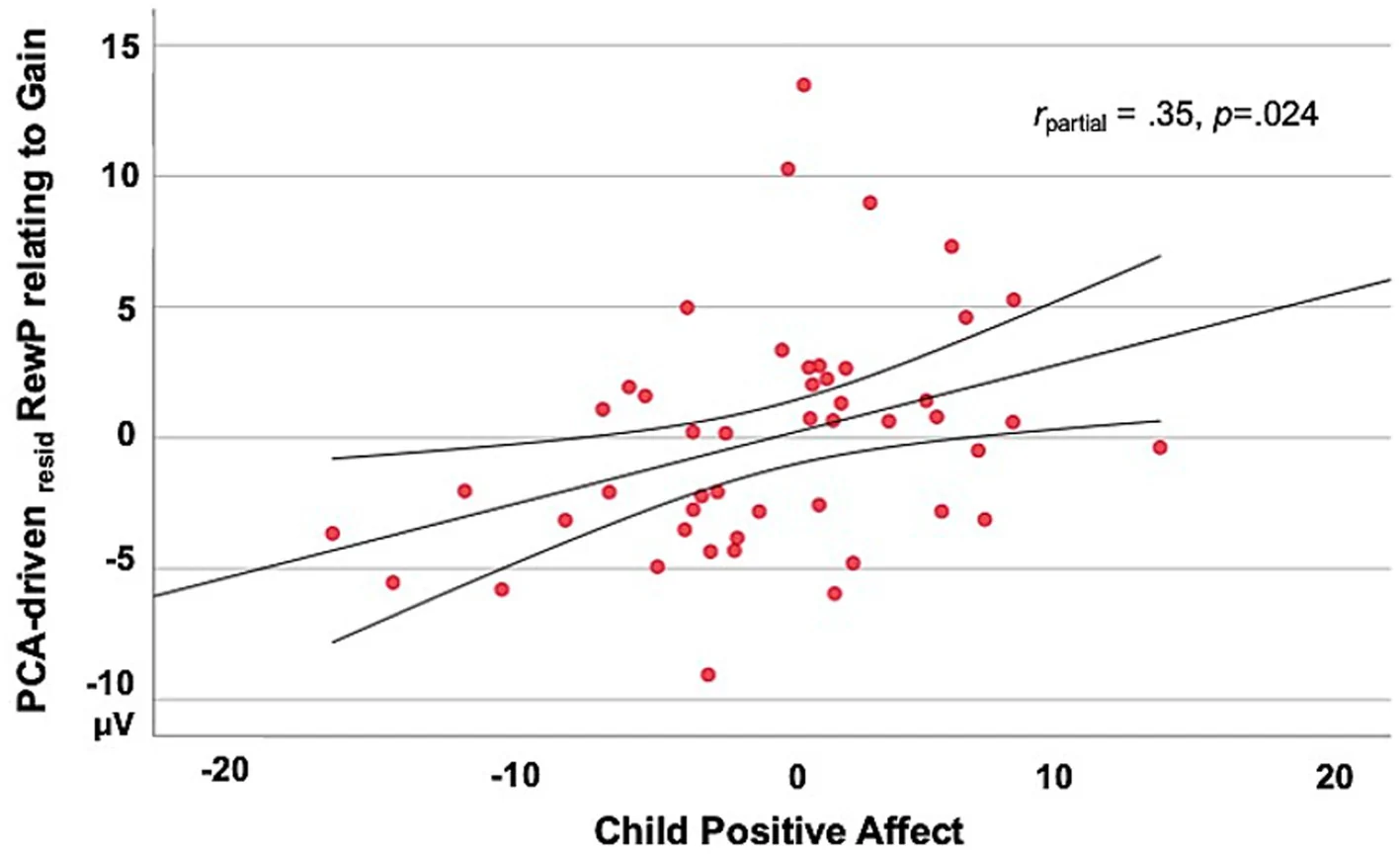
Partial regression plot showing the association between individual differences in child positive affect and PCA-driven RewP ERP relating gain feedback. Figure presents a partial regression plot from the final hierarchical regression model (*n = 4*8). The plot depicts the unique association between child positive affect and PCA-derived residualized RewP after controlling for child age, sex, ethnicity, parental depressive symptoms, child depressive symptoms, and income-to-needs ratio. Each point represents residualized values after accounting for the covariates included in the model. The central line represents the fitted regression line, and the curved lines indicate the 95% confidence interval. The partial correlation coefficient and corresponding *p*-value are displayed in the figure. *X*-axis: Positive affect was assessed using the Positive Affect subscale of the Positive and Negative Affect Schedule for Children (PANAS-C; Laurent et al., 1999).

### 3.5 Sensitivity analyses

Because four participants retained fewer than 10 artifact-free trials in at least one condition (either Reward or loss conditions), an additional sensitivity analysis was conducted excluding these participants. The pattern of findings remained unchanged. Specifically, child positive affect continued to significantly predict PCA-derived RewP amplitude after controlling for child age, ethnicity, income-to-needs ratio, parental depressive symptoms, child depressive symptoms, and sex (*β* = 0.43, *p* = 0.02), whereas child negative affect was not significantly associated with PCA-derived RewP (*p* = 0.65). These findings suggest that the observed association between positive affect and RewP was not driven by participants with low trial counts.

Because income-to-needs ratio data were available for only a subset of participants (*n* = 48), a second sensitivity analysis was conducted excluding this covariate to maximize sample size. In this larger sample, child negative affect significantly predicted PCA-derived RewP amplitude after controlling for child age, ethnicity, parental depressive symptoms, child depressive symptoms, and sex (*β* = 0.26, *p* = 0.01), whereas child positive affect was not significantly associated with PCA-derived RewP (*β* = 0.07, *p* = 0.48). Thus, although associations between affective functioning and RewP were observed across analytic models, the specific affective predictor varied depending on the inclusion of income-to-needs ratio.

To evaluate potential selection bias associated with missing income-to-needs ratio data, participants with available income-to-needs data were compared with those missing income-to-needs data. The groups did not differ significantly in child positive affect, *t*(112) = 1.19, *p* = 0.24, child negative affect, *t*(112) = 0.35, *p* = 0.73, child depressive symptoms, *t*(112) = −0.71, *p* = 0.48, parental depressive symptoms, *t*(113) = 0.55, *p* = 0.59, PCA-derived P200 amplitude, *t*(111) = −0.65, *p* = 0.52, or PCA-derived RewP amplitude, *t*(110) = −0.20, *p* = 0.84. In addition, participants with and without income-to-needs data did not differ significantly in sex, χ^2^(1, *N* = 114) = 0.52, *p* = 0.47, ethnicity, χ^2^(1, *N* = 113) = 0.31, *p* = 0.58, or racial composition, χ^2^(4, *N* = 113) = 4.52, *p* = 0.34. However, participants with available income-to-needs data were significantly older than those without income data (*M* = 76.57 months, *SD* = 10.02 vs. *M* = 69.46 months, *SD* = 11.60), *t*(132) = 3.57, *p* < 0.001. Thus, the analytic subsample used in the primary regression analyses represented an older subset of the overall sample. Although age was included as a covariate in all regression models, statistical adjustment does not fully eliminate potential effects of systematic differences in sample composition.

### 3.6 Exploratory analyses of the temporal stability of the RewP

To evaluate the temporal stability of reward-related neural responses, exploratory analyses were conducted in a subset of participants who completed the Doors Guessing Task approximately 1 year later (*n* = 43). Baseline and follow-up residualized RewP scores were significantly correlated, *r* = 0.33, *p* = 0.03. Consistent with this finding, a two-way mixed-effects intraclass correlation coefficient (ICC; consistency definition) indicated modest but significant one-year stability, ICC(3,1) = 0.32, 95% CI [0.03, 0.57], *p* = 0.01. Given the one-year interval during early childhood, this estimate should be interpreted cautiously, as the observed variability likely reflects a combination of developmental change, measurement variability, and other within-subject factors that cannot be disentangled in the present study. Accordingly, these analyses provide only a limited assessment of the temporal stability of the residualized RewP.

## 4 Discussion

The present study examined whether individual differences in reward-related neural responses are associated with normative emotional functioning during early childhood, a critical developmental window for affective and motivational systems (Gunnar and Quevedo, 2007; Tottenham, 2015). Using temporospatial PCA to isolate feedback-related ERPs in children aged 4–7 years, we found that, consistent with our hypothesis, higher positive affect was associated with larger PCA-derived Reward Positivity (RewP) amplitudes, even after controlling for demographic factors and both child and parental depressive symptoms. In contrast, sex differences were observed in the PCA-derived P200, with boys showing greater differentiation between gain and loss feedback than girls. This pattern suggests that sex-related variability in this age range may emerge more strongly at earlier stages of attentional allocation to motivationally salient feedback, whereas positive affect may be more closely tied to later reward-evaluation processes indexed by the RewP. However, because the PCA-derived reward-related component identified in the present study differs from the canonical RewP typically reported in older children, adolescents, and adults, its functional interpretation should be made cautiously. Although we refer to this factor as the RewP throughout for consistency with prior literature, it remains uncertain whether it reflects the same underlying neurocognitive construct or instead represents a developmentally distinct balance of reward- and loss-related processing during early childhood. Given the limited literature on positive affect and feedback-related P200 activity in early childhood, these findings should be interpreted as preliminary but suggest that temporospatial PCA can reveal developmentally meaningful distinctions between early attentional and later reward-related components.

### 4.1 RewP ERP and positive valence functioning in early childhood

Within the recent NIMH’s RDoC framework, the Positive Valence Systems domain encompasses neural and behavioral processes involved in reward responsiveness, reward learning, and approach motivation (Cuthbert et al., 2023). In adolescents and adults, RewP has been robustly linked to reward sensitivity, hedonic capacity, and individual differences in approach-related affect, and blunted RewP has been associated with depression and anhedonia (Brown et al., 2022; Pizzagalli, 2014; Proudfit, 2015). The current findings extend this literature to early childhood by demonstrating that individual differences in positive affect are associated with variability in reward-related neural responding among children ages 4–7 years. Furthermore, the association between positive affect and the PCA-derived RewP remained significant after controlling for both child depressive symptoms and parental depressive symptoms. This finding suggests that the observed relation was not solely attributable to variation in depression-related symptomatology or familial risk factors, but rather reflects a unique association between positive emotional functioning and neural responsiveness to reward. Notably, neither child depressive symptoms nor parental depressive symptoms were significantly associated with the PCA-derived RewP in the present sample. Although numerous studies have reported reduced RewP amplitudes among individuals with depression or elevated depression risk (e.g., Proudfit, 2015), findings have been mixed, particularly in young children and community samples. The absence of a significant association in the present study may reflect the relatively low levels and restricted variability of depressive symptoms in this normative sample, suggesting that individual differences in positive affect may be more closely related to reward responsiveness than concurrent depressive symptoms during early childhood. Although reduced RewP amplitudes have frequently been linked to depression and depression risk, particularly anhedonic symptoms (Proudfit, 2015), positive affect represents a related but distinct construct that captures normative individual differences in reward engagement, approach motivation, and the tendency to experience positive emotions. Our findings therefore extend prior work by demonstrating that greater positive affect is associated with enhanced reward-related neural activity even after accounting for concurrent depressive symptoms.

The present findings are broadly consistent with studies demonstrating that larger RewP amplitudes are associated with greater reward responsiveness, positive emotionality, and approach-related functioning in adolescents and adults (Angus et al., 2015; Duttweiler et al., 2024). Extending this literature, the current findings suggest that individual differences in positive affect are associated with reward-related neural responding as early as ages 4–7 years. More broadly, these findings align with the RDoC emphasis on dimensional constructs spanning normal and abnormal functioning and underscore the value of examining reward-related neural processes in relation to positive emotional traits during sensitive developmental periods (Nusslock and Alloy, 2017).

An important consideration when interpreting these findings is that the PCA-derived reward-related factor differed from the canonical adult RewP in both latency and polarity. The identified factor peaked at approximately 392 ms and exhibited a frontocentral negative-going morphology, whereas the adult RewP is typically characterized as a relative positivity to rewards peaking between 250 and 350 ms. However, developmental studies indicate that reward-related ERP responses in young children often exhibit broader morphologies, delayed peak latencies, and substantial temporal overlap with adjacent components, including the P300 (Belden et al., 2016; Hennefield et al., 2022; Lukie et al., 2014). Consistent with this interpretation, temporospatial PCA indicated that the RewP (TF1/SF1) and P300 (TF1/SF2) emerged from the same temporal factor but were distinguished by separate spatial factors. The RewP factor showed a frontocentral distribution and significantly differentiated gain and loss outcomes, whereas the P300 factor showed a centroparietal distribution and did not significantly differentiate feedback valence. Accordingly, we interpret this factor as a reward-sensitive ERP component broadly consistent with the RewP literature while acknowledging that direct comparisons with conventionally scored RewP measures from older developmental samples and adults should be made cautiously.

Although the PCA-derived factor exhibited negative-going responses to both gain and loss feedback, the critical feature defining this component was the relative differentiation between reward and loss outcomes, with gain eliciting a less negative response than loss. Thus, the designation “RewP” is based on the direction of the gain–loss contrast rather than the absolute polarity of the PCA factor. Because PCA extracts latent components whose polarity is mathematically arbitrary and whose amplitudes reflect weighted combinations of overlapping neural sources, the absolute polarity of the factor should not be interpreted in the same manner as conventionally scored ERP waveforms (Dien, 2012; Foti et al., 2011a). Nevertheless, we agree that the more pronounced absolute response to loss relative to gain distinguishes the present PCA-derived factor from several PCA-derived RewP components reported in older children and adolescents (e.g., Ethridge and Weinberg, 2018; Kujawa et al., 2018). This difference may reflect developmental changes in reward-related neural processing, greater temporal overlap among feedback-related ERP components in early childhood, or differences in task design and PCA decomposition. Additional developmental work will be needed to determine whether this pattern is specific to early childhood or reflects methodological differences across studies. More importantly, it remains uncertain whether the present PCA-derived factor is construct-equivalent to the canonical RewP described in older developmental samples and adults. Although the factor differentiated gain from loss outcomes in the expected direction and was associated with positive affect, its atypical morphology and relatively larger loss-related response suggest that it may represent a developmentally distinct manifestation of reward-related processing or a different balance between reward- and loss-related neural activity. Accordingly, comparisons with the broader RewP literature should be made cautiously until future developmental studies directly establish the functional equivalence of these measures.

### 4.2 Affective correlates of the RewP

Although the primary analyses indicated that RewP was associated with positive affect but not negative affect, sensitivity analyses excluding income-to-needs ratio yielded a somewhat different pattern, with negative affect emerging as a significant predictor of RewP amplitude. Thus, the observed associations between affective functioning and reward-related neural responses appear sensitive to model specification and the inclusion of socioeconomic covariates. Nevertheless, the primary analyses suggest that positive affect may be more closely associated with reward-related neural responding when socioeconomic context is taken into account, consistent with theoretical models linking reward responsiveness to positive valence functioning. Similarly, neither child depressive symptoms nor parental depressive symptoms were significantly associated with the PCA-derived RewP in the present sample. Although this finding differs from studies of clinically depressed or high-risk populations, it is consistent with the possibility that, within a community sample of young children, RewP variability is more strongly related to normative individual differences in positive emotional functioning than to concurrent depressive symptom levels. Although positive and negative affect are moderately related, developmental evidence suggests that they reflect partially separable dimensions of emotional functioning, with positive affect showing distinct associations with approach motivation, reward responsiveness, and resilience, and negative affect showing stronger links with distress, threat sensitivity, and internalizing symptoms (Dennis et al., 2009; Forbes and Dahl, 2005; Hanson et al., 2021; Olino et al., 2011; Schreiber and Hallquist, 2026). The selective association with positive affect observed here is consistent with models positing that RewP primarily reflects neural responses to rewarding outcomes and approach-related motivation rather than general emotional reactivity (Brown et al., 2022; Proudfit, 2015). These findings contribute to a growing body of work suggesting that reward-related ERPs are more closely tied to hedonic and motivational dimensions of affect than to negative emotionality per se (Jackson and Cavanagh, 2023; Renault et al., 2023).

Importantly, this specificity has implications beyond normative emotional functioning. If, as suggested by our primary analyses, the RewP primarily indexes individual differences in positive valence functioning, then attenuated reward responsiveness may reflect reduced engagement with positive environmental stimuli, potentially increasing vulnerability to depressive symptoms by diminishing opportunities for positive reinforcement. From a developmental psychopathology perspective, this distinction is particularly important because reduced positive affect and diminished reward responsiveness have been proposed as early emerging vulnerability markers for depression (Belden et al., 2016; Gilbert et al., 2021; Luking et al., 2021). Converging behavioral and neurophysiological evidence suggests that alterations in reward processing are detectable well before adolescence (Fowler and Gaffrey, 2022; Gaffrey et al., 2018b; Luby et al., 2014). Notably, studies of young children in the preschool to early school-age range (approximately 4–7 years) have demonstrated blunted neural responses to reward, indexed by a reduced reward positivity (RewP), in children with depressive symptoms or early-onset depression (Belden et al., 2016). These findings indicate that disruptions in reward-related neural processing are already evident during a developmental period characterized by rapid maturation of affective and motivational systems.

Early childhood is also a critical window for the normative development of positive affect, approach motivation, and sensitivity to reward feedback. Therefore, identifying whether diminished RewP in 4–7-year-olds reflects disrupted reward evaluation, altered early attentional engagement (e.g., P200-related processes), or both has significant implications for understanding mechanisms underlying early depressive risk. Clarifying the functional specificity of reward-related ERP components in this age range may help differentiate generalized reductions in positive emotional reactivity from more targeted impairments in reward processing, thereby sharpening developmental models of depression vulnerability. By demonstrating that RewP is associated with positive affect—but not negative affect—during early childhood, the present findings provide novel evidence that individual differences in reward processing may shape trajectories of positive emotional functioning well before clinically significant symptoms emerge.

In contrast to the RewP, the PCA-derived P300 did not significantly differentiate gain and loss outcomes and therefore was not included in subsequent regression analyses. The P300 is generally thought to reflect attentional allocation, context updating, and outcome evaluation processes rather than reward responsiveness per se (Polich, 2007; Wu and Zhou, 2009). The absence of a significant P300 effect, coupled with the observed association between RewP and positive affect, further supports the functional specificity of the RewP as a neural marker of reward-related positive valence functioning (Bress and Hajcak, 2013; Proudfit, 2015). It is also possible that developmental characteristics of feedback-related ERP responses in early childhood contributed to the lack of a distinct P300 effect. Young children exhibit substantial temporal overlap among adjacent ERP components and delayed component latencies relative to older children and adults (Crowley et al., 2009; de Haan, 2007; Lukie et al., 2014). Temporospatial PCA was specifically employed to disentangle these overlapping neural processes, enabling the isolation of the reward-sensitive RewP from adjacent components, including the P300 (Dien, 2012; Foti et al., 2011a). Taken together, the present findings indicate that individual differences in affective functioning are associated with reward-related neural responses during early childhood. Although the specific affective predictor varied across analytic models, the results collectively suggest that variability in RewP may reflect meaningful individual differences in emotional functioning during a developmental period characterized by rapid maturation of affective and motivational systems.

### 4.3 Sex differences in gain-related P200

In contrast to the RewP findings, sex differences emerged for the PCA-derived P200, with boys exhibiting greater amplitudes than girls. The P200 is often interpreted as reflecting early attentional allocation to salient stimuli, including reward-related cues (Kiat et al., 2015; Vartak et al., 2017; Xin et al., 2021; Zou et al., 2022). Consistent with prior work in adults (Jin et al., 2013; Torrence and Troup, 2018), sex differences in P200 amplitude may therefore reflect differences in early-stage processing or attentional engagement with rewarding outcomes, rather than differences in reward evaluation per se. Notably, sex differences emerged for the P200 but not for the RewP at the group level, suggesting that early attentional processes and later evaluative processes may follow distinct sex-related developmental trajectories. These findings should be interpreted cautiously, as sex differences in feedback-related ERPs during early childhood remain understudied (Belden et al., 2016). Nonetheless, they raise the possibility that sex-linked differences in attentional engagement with reward may emerge early in development and could interact with socialization or environmental factors over time. Longitudinal studies will be essential for clarifying whether such differences persist, diminish, or intensify across development. Importantly, efficient attentional allocation to rewarding or reward-predicting stimuli is a critical component of reward-based learning (Gui et al., 2016; Tibboel and Liefooghe, 2020; Vartak et al., 2017) and reduced P200 amplitudes have been linked to learning difficulties across academic domains in children (Chao et al., 2022; Fernández et al., 2014). Given that disrupted reward-based learning processes are implicated in several neurodevelopmental and psychiatric conditions that show marked sex differences in prevalence and presentation (Belden et al., 2016; Forester et al., 2022; Landes et al., 2018; Thoma et al., 2015), the observed sex differences in P200 responses may have meaningful implications for understanding early-emerging pathways of risk. Future work should directly test whether the P200 contributes to reward-driven learning behaviors in a sex-specific manner and whether early differences in attentional engagement with reward predict divergent developmental outcomes.

### 4.4 Methodological implications: advantages of PCA in pediatric ERP research

A key methodological contribution of this study is the use of temporospatial PCA to isolate reward-related ERP components in young children. Interpretation of ERPs in early childhood is complicated by overlapping components, increased neural variability, and developmental differences in waveform morphology (Brooker et al., 2020). By applying PCA, we were able to disentangle the RewP from overlapping components, including the P200 and P300, thereby enhancing construct validity and interpretability. The observed associations with PCA-derived RewP amplitude and positive affect, together with the absence of comparable findings for other components, further supports the utility of PCA-derived measures as developmentally meaningful indices of reward processing.

Importantly, additional analyses provided evidence for both the robustness and preliminary reliability of the PCA-derived RewP. The association between positive affect and RewP remained significant after excluding participants with fewer than 10 artifact-free trials in at least one condition, indicating that the findings were not driven by low trial accounts or data quality concerns. This is particularly relevant in pediatric ERP research, where obtaining large numbers of artifact-free trials can be challenging. Furthermore, exploratory analyses revealed modest but significant one-year stability of the PCA-derived RewP, as reflected by both a significant correlation between baseline and follow-up scores and a significant intraclass correlation coefficient. Although the magnitude of stability was modest, there findings are consistent with expectations for neural measures collected during a period of rapid developmental change and suggest that individual differences in reward responsiveness can be detected reliably across time.

Taken together, these findings expand prior methodological work by demonstrating not only that temporaospatial PCA can improve the separation of overlapping ERP components in young children, but also that the resulting RewP measure shows meaningful associations with affective functioning, robustness to variations in trial counts, and preliminary temporal stability. These findings highlight the importance of using analytic approaches that are sensitive to developmental differences in neural signals (Barry et al., 2022; Gibb et al., 2022) and suggest that PCA-based methods may be particularly well suited for studying reward processing in early childhood.

### 4.5 Limitations and future directions

Several limitations should be considered. First, the modified doors task used in this study, which is commonly employed in pediatric reward-processing research (Belden et al., 2016; Hennefield et al., 2022), focused on reward receipt and feedback evaluation and did not include a reward anticipation phase. This is an important limitation, as growing evidence suggests that anhedonia - a key endophenotype of depression- may be more strongly linked to deficits in reward anticipation than to feedback processing per se (Chen et al., 2018; Pizzagalli, 2014; Zhou et al., 2019). Consequently, the present findings cannot determine whether sex-specific differences in positive affect are also evident during anticipatory stages of reward processing in early childhood. Although the modified Doors task included neutral feedback trials, the present study focused on the conventional gain-versus-loss comparison to isolate reward-related neural responses (RewP), consistent with the predominant RewP literature. The neutral feedback condition served as a control condition and was not intended as an analytic reference for quantifying the RewP. Moreover, the neutral feedback (“--”) differed perceptually from the gain and loss feedback, which consisted of Skittles images, making gain–neutral and loss–neutral comparisons difficult to interpret because observed differences could reflect both perceptual and reward-related processes. Consequently, the present study was not designed to distinguish the relative contributions of reward- and loss-related neural activity to the observed RewP effects. Future studies employing perceptually matched neutral feedback conditions would help further clarify the respective contributions of reward- and loss-related processes during early childhood. Although recent work has emphasized subject-level ERP reliability and data-quality metrics such as RMS(SME) (Luck et al., 2021; Clayson et al., 2017), these metrics could not be retrospectively computed because only condition-averaged PCA-derived ERP data were retained for the present analyses. Future studies should preserve trial-level data to permit more comprehensive psychometric evaluation of both conventional and PCA-derived ERP measures. Nevertheless, exploratory analyses indicated relatively low one-year temporal stability of the residualized RewP. Given the one-year interval during early childhood, this estimate likely reflects a combination of developmental change and measurement variability and therefore should be interpreted cautiously.

Second, an important limitation concerns missing socioeconomic data. Income-to-needs ratio was available for only a subset of participants, resulting in a smaller sample for the primary regression analyses. Participants with and without income-to-needs data did not differ significantly in sex, race, ethnicity, affective functioning, depressive symptoms, or ERP measures, suggesting that the analytic subsample was broadly comparable to the larger sample on most measured characteristics. However, participants with available income-to-needs data were significantly older than those without these data. Although age was included as a covariate in all regression models, statistical adjustment does not fully eliminate potential consequences of systematic differences in sample composition. Given the rapid developmental changes that occur during early childhood, age-related differences may be associated with variation in reward-related behavior, ERP morphology, or other aspects of neural development that are not fully captured by linear covariate adjustment. Sensitivity analyses excluding income-to-needs ratio yielded a somewhat different pattern of associations, with negative affect rather than positive affect emerging as a significant predictor of RewP amplitude. Because socioeconomic status has been linked to both emotional development and reward-related neural functioning (Granros et al., 2024; Murray et al., 2020; Taylor et al., 2017; Yaple and Yu, 2020), the primary analyses retained income-to-needs ratio as a theoretically important covariate in the primary analyses despite the resulting reduction in sample size. Accordingly, the primary findings should be interpreted as reflecting the subset of participants with complete socioeconomic data and should be replicated in larger samples with more complete socioeconomic information to establish their generalizability.

Third, the primary analyses were cross-sectional, limiting inferences regarding developmental continuity or change. It remains unclear whether the sex-related effects observed here are specific to early childhood or generalize to later developmental periods, such as middle childhood or adolescence. However, the present sample was drawn from a larger longitudinal study of neural reward processing and emotional development, and exploratory analyses indicated modest but significant one-year stability of the RewP. These findings provide preliminary evidence that individual differences in reward-related neural responding can be detected across time during early childhood. Future analyses leveraging the broader longitudinal design will be important for examining developmental trajectories of reward processing and determining whether early neural responses to reward predict subsequent changes in positive affect, depressive symptoms, or other indicators of emotional functioning. Longitudinal designs spanning key developmental transitions, including puberty, will be also essential for determining whether early individual differences in reward-related feedback processing predict later alterations in reward-related neural function or psychopathology risk.

Fourth, the present study focused on normative variation in affective functioning and did not include a clinical sample. Although the observed association between RewP and positive affect is consistent with theoretical models linking reward responsiveness to depression vulnerability, it remains unclear whether these findings generalize to children with clinically significant depressive symptoms or diagnosed depressive disorders. Future studies should directly examine reward-related ERP components in clinical and at-risk pediatric populations to determine whether the associations observed here reflect normative individual differences in positive valence functioning, early markers of depression vulnerability, or both. Such work may help clarify the clinical significance of reward-related neural processes during early childhood and inform developmental models of affective psychopathology.

Lastly, the generalizability of these findings may be constrained by limited demographic diversity. Socioeconomic status, environmental stress exposure, and access to enriching experiences are known to influence reward processing and emotional development. Larger and more socioeconomically diverse samples may reveal different patterns of sex differences or sex-specific associations between neural indices of reward processing (e.g., P200, RewP/FN) and affect. Addressing these limitations will be essential for refining developmental models of sex-specific reward processing and their relevance to affective psychopathology.

## 5 Conclusion

In sum, this study provides novel evidence that PCA-derived RewP is associated with individual differences in affective functioning during early childhood. In primary analyses that accounted for socioeconomic status and other relevant covariates, positive affect emerged as a significant predictor of RewP amplitude, supporting the relevance of reward-related neural responses to positive valence functioning within the RDoC Positive Valence Systems framework. By extending reward-processing research to an understudied developmental period and applying temporospatial PCA to isolate reward-related neural activity, the present findings highlight the potential utility of this PCA-derived reward-related ERP component as an early neural correlate of affective functioning. However, additional developmental work is needed to determine the extent to which this component is functionally equivalent to the canonical RewP reported in older children, adolescents and adults. More broadly, the results suggest that individual differences in reward-related neural responding are evident during early childhood and may contribute to variation in affective development. Future longitudinal studies are needed to clarify how reward-related neural processes change across development and whether early individual differences in RewP predict subsequent reward-related behaviors, emotional functioning, and risk for psychopathology.

## Data Availability

The original contributions presented in the study are included in the article/Supplementary material, further inquiries can be directed to the corresponding author.
