# Supplementary material for: Reward positivity and affective functioning in early childhood: evidence from temporospatial principal components analysis
Source: Front Hum Neurosci. 2026 Oct 7;20:1839910. doi: 10.3389/fnhum.2026.1839910 (PMC13683569; doi:10.3389/fnhum.2026.1839910)
Supplement: Supplementary file 1 [file Data_Sheet_1.docx]

**Supplemental Materials**

**S1. METHODS AND MATERIALS**

**S1.1. Inclusion and exclusion criteria**

Participants were drawn from a larger longitudinal study examining reward processing as an early-emerging risk factor for depression. Eligible caregivers were required to be the child’s biological mother, serve as the primary caregiver, and have resided with the child for at least the previous six months. Children were required to be between 4 and 7 years of age at the time of their first laboratory visit. Children were excluded if they (1) were born preterm (<35 weeks gestation); (2) were exposed prenatally to alcohol, illicit drugs, or tobacco; (3) had a history of seizures, loss of consciousness, brain swelling, or hospitalization due to head injury; (4) had been diagnosed with hydrocephalus, hypoxic–ischemic encephalopathy, cerebral palsy, brain tumor, genetic disorder, hearing or vision impairment, chronic illness, attention-deficit/hyperactivity disorder, or a pervasive developmental disorder; (5) exhibited speech, language, or other developmental delays; (6) had a medical condition associated with behavioral concerns; or (7) were currently taking medications for mood, anxiety, or attention-related difficulties. Caregivers completed the short form of the Preschool Feelings Checklist (PFC-Short; (Luby et al., 2004) to assess children’s depressive symptoms. Consistent with the primary aims of the parent study, children with low (≤1 item endorsed) or high (≥3 items endorsed) levels of depressive symptoms were included, whereas children with moderate levels of depressive symptoms (2 items endorsed) were excluded; the same criteria were applied in the present study.

**S1.2. Detailed EEG Preprocessing Steps**

Because EEG data collection occurred during the COVID-19 pandemic, participants were required to wear face masks during EEG sessions in accordance with Duke University COVID-19 safety policies. Consequently, 24 channels located on the outer ring of the sensor net were removed from analyses because their proximity to the face and neck resulted in excessive artifacts.

Additional preprocessing details beyond those described in the main manuscript are provided here. Data segments without relevant events (e.g., breaks between task blocks) were removed prior to preprocessing. All data were re-referenced to the average of the mastoids. Bad channels were identified using the Clean Rawdata plugin and were removed if they (1) were flat for more than five seconds, (2) contained more than four standard deviations of line noise relative to the signal, or (3) correlated less than .80 with nearby channels.

To optimize independent component analysis (ICA) performance, a copy of the data was created and high-pass filtered at 1 Hz (4th-order Butterworth filter) using EEGLAB v2021 (Delorme & Makeig, 2004). Artifact Subspace Reconstruction (ASR; (Chang et al., 2020) was then applied using a burst criterion of 20 (Chang et al., 2020). Additional data segments were flagged as bad when the power of any channel exceeded seven standard deviations. A segment was rejected only when more than 25% of channels within that segment were identified as bad.

Extended infomax ICA (Lee et al., 1999) with PCA dimension reduction to 50 components was performed on the filtered copy of the data. The resulting ICA matrix was then transferred to the original full-length dataset (i.e., the dataset prior to ASR-based segment removal). Eye-related components were automatically identified and removed using the ICLabel plugin (Pion-Tonachini et al., 2019), with a probability threshold of .70. Applying the ICA solution to the original dataset allowed preservation of data that would otherwise have been discarded during artifact correction procedures. Finally, channels previously identified as bad were interpolated using spherical spline interpolation.

Supplemental Figure 1. 128-channel Nets showing Region of Interest

**References for the Supplementary Materials**

Chang, C. Y., Hsu, S. H., Pion-Tonachini, L., & Jung, T. P. (2020). Evaluation of Artifact Subspace Reconstruction for Automatic Artifact Components Removal in Multi-Channel EEG Recordings. *IEEE Trans Biomed Eng*, *67*(4), 1114–1121. <https://doi.org/10.1109/tbme.2019.2930186>

Delorme, A., & Makeig, S. (2004). EEGLAB: an open source toolbox for analysis of single-trial EEG dynamics including independent component analysis. *J Neurosci Methods*, *134*(1), 9–21. <https://doi.org/10.1016/j.jneumeth.2003.10.009>

Lee, T. W., Girolami, M., & Sejnowski, T. J. (1999). Independent component analysis using an extended infomax algorithm for mixed subgaussian and supergaussian sources. *Neural Comput*, *11*(2), 417–441. <https://doi.org/10.1162/089976699300016719>

Luby, J. L., Heffelfinger, A., Koenig-McNaught, A. L., Brown, K., & Spitznagel, E. (2004). ThePreschool Feelings Checklist: a brief and sensitive screening measure for depression in young children. *J Am Acad Child Adolesc Psychiatry*, *43*(6), 708–717. <https://doi.org/10.1097/01.chi.0000121066.29744.08>

Pion-Tonachini, L., Kreutz-Delgado, K., & Makeig, S. (2019). ICLabel: An automated electroencephalographic independent component classifier, dataset, and website. *Neuroimage*, *198*, 181–197. <https://doi.org/10.1016/j.neuroimage.2019.05.026>
